# Report of the Proceedings of the Illinois State Board of Health

**Published:** 1887-12

**Authors:** 


### REPORT OF THE PROCEEDINGS OF THE ILLINOIS STATE BOARD OF HEALTH.

QUARTERLY, MEETING, CHICAGO, OCTOBER 18, 1887.

The regular quarterly meeting of the Illinois State Board of Health was held in this city on October 18, 1887.

Drs. W. A. Haskell, President, Newton Bateman, A. L. Clark, W. R. Mackenzie, H. V. Ferrell and John H. Rauch, Secretary, were present.

The Secretary presented the following

QUARTERLY REPORT.

During the quarter ended September 30, 1887, there were received in the Secretary's office 1,798 written communications of all kinds, and a total of 1,050 were sent out. Of printed matter there were distributed 143 copies of the various Annual Reports, 47 Official Registers, 24 copies of Report on Medical Education, 7,200 copies of Report of Proceedings of July, 1887, Meeting of the Board,—which contained also the new law regulating the practice of medicine, in force July 1, 1887,—and the usual number of the various publications of the Board, preventable disease circulars, etc.

CERTIFICATES AND LICENSES.

There were issued 103 certificates to physicians during the quarter; 84 to graduates upon diplomas from medical colleges in good standing; 1 upon examination; 14 to non-graduates upon proof of ten years' practice in the State prior to July 1, 1877, and 4 duplicates upon proof of destruction of the originals. Certificates were also issued to twenty-five midwives; 10 upon diplomas or licenses, 12 upon examination and three upon years of practice.

The unusually large number of certificates issued—greater than ever before during the third quarter of any calendar year since the Board was organized—is due to the operation of the new medical practice act, which has brought all physicians under the provisions of the law, without regard to length of practice in the State. Of the 103 certificates issued no less than 38 were to practitioners previously exempt—24 to graduates and 14 to non-graduates who were not required to take out certificates under the act of 1877. More applications for certificates (60 in all) have also been deferred for the action of the Board, or definitely rejected, than during any corresponding period since 1878. This result was to be expected, since the great majority of skillful, qualified physicians had complied with the law of 1877, and were already duly certificated. While there were a number of physicians of the same reputable character and attainments as these in the small minority remaining, there were many of a very different class, who were exempted from supervision by the ten-years' prior-practice clause of the old law. These are now required to hold the certificate of the Board as a guaranty of their moral and professional fitness to practice medicine, and it is mainly from these that the rejected or deferred applications have come. These applications have been deferred or rejected because the applicants are unable to comply with the law.

THE NEW MEDICAL PRACTICE ACT.

Although entailing an immense amount of detail labor, and giving rise to many perplexing questions, the results of the operation of the new act to regulate the practice of medicine thus far have been fairly gratifying. A number of suits for violation of its provisions have been brought locally against quacks and other disreputables. In eleven of these the suits have been pushed to a successful conclusion.

One of the most signal benefits of the new law has been already demonstrated in practically ridding the State of the itinerant nostrum venders. At the close of the quarter there were only two of this class of charlatans left in the State, and the necessary proceedings have been already begun to secure their conviction or compel them to abandon Illinois as a field for their efforts.

MEDICAL EDUCATION.

The revision of the Report on Medical Education is almost perfected, and the tables and conclusions will be submitted for the information of the Board during the meeting. A period of ten years is now covered by this report, and the data concerning 258 medical colleges of all classes and an aggregate of over 116,000 medical students, including some 37,000 graduates, are embraced in its successive editions.

One of the most striking features elicited by this research and its mass of facts and figures is the cumulative evidence afforded of the influence of the action of this Board upon the subject of medical education throughout the United States. In 1880 the Board announced its adoption of a Schedule of Minimum Requirements, compliance with which schedule should be the condition of "good standing" of any given medical college after the sessions of 1882–83. At the former date, 1880, the percentage of graduates to matriculates was about 35; it has now fallen to 30.5. The improvement is even more marked in the regular schools in which during the sessions of 1881–82, immediately following the announcement of the Board, the proportion of graduates to matriculates was 35.8 per cent. During the last sessions this percentage was 28.8—showing an improvement, numerically expressed, of over 18 per cent. in this respect.

These changes are still more significant when it is considered that the exaction of a preliminary education and the longer term of study, attendance on clinics, laboratory work, etc., have excluded, on the one hand, a large number of those primarily unfit for the study of medicine, and, on the other hand, have given the graduates a more thorough training and higher qualifications for the practice of their profession. The extent to which this process of exclusion of the unfit has been carried is shown in the total numbers of those engaged in the study of medicine in 1882–83 and in 1886–87, respectively. During the first-mentioned period there were 13,088 matriculates reported at the various colleges. At the last sessions, notwithstanding the growth of population, there were only 12,939 in attendance. Other figures show equally gratifying improvement in other directions.

THE PUBLIC HEALTH.

Since the last meeting of the Board, the matter of greatest moment to health officials and health boards everywhere in this country has been the arrival of the cholera-infected steamer *Alesia* at the port of New York on September 25th. With the general facts concerning this arrival, and the action of the New York quarantine officers in the premises, the Board and the public have been advised by the daily press. It is too early to assert that all danger is now past from this arrival. So far as the developments go, the occurrence is a sufficient justification of the action of the Board in empowering its secretary, more than two years ago, to make an inspection of the quarantines maintained on the Atlantic and Gulf coasts with the especial reference to the exclusion of Asiatic cholera and small-pox. In the introduction to that report, made early in 1886, it was said that "these outer lines, even with some defects and weak places, I believe to be even now sufficiently strong to keep out the disease if proper vigilance and thoroughness be exercised, if all the facilities be utilized, and if timely notification of threatened danger be given by the National Government.

"The next year or two, however, will furnish a tolerably conclusive test of the efficiency of quarantine to exclude the pestilence from this country."

More than a year before this report, in an address at the opening of the National Conference of State Boards of Health, held in St. Louis, October 13, 1884, it was urged that "Congress should give the President the power to issue a proclamation, upon the recommendation of the National health authority, forbidding immigration into the United States from infected districts of other countries, and it should provide some method of international sanitary co-operation between this country and the Dominion of Canada, whose interests are substantially the same as ours in these matters, and whose contiguity makes co-operation of vital importance."

These important matters are yet neglected, and there is not any provision for timely notification of threatened danger by any National authority.

The *Alesia* arrived at a port where the quarantine regulations require every foreign arrival to be boarded and inspected before allowing the vessel to proceed to her dock. The existence of the cholera infection was thus promptly ascertained on this vessel, and the proper steps were at once taken to segregate the sick from the well, to place these latter in a quarantine of observation, and to isolate the former so as to prevent any spread of the disease from existing cases. Meanwhile approved disinfecting and cleansing processes were applied to the vessel, to the dunnage and personal effects of the crew and passengers, and to other material susceptible of being fomites.

That these precautions were imperatively necessary is shown by the continued development for fully two weeks of new cases among the apparently healthy isolated on Hoffman's island. Notwithstanding all the precautions which were observed, the disease continued to attack people removed as far as possible from all known sources of infection during a period of fourteen days.

Can it be doubted that, had the English theories of cholera and of quarantine obtained in New York harbor during the past few weeks—can it be doubted that these twenty-six cases or a large proportion of them would have been scattered throughout the country, each one to form a possible epidemic center ? It is, of course, this consideration which excites our interest in the administration of the coast quarantines.

Dr. W. M. Smith (health officer at the port of New York) is believed to have been prompt, explicit and candid in his telegraphic statements, and this belief has reassured the whole country. The interior has assumed that everything known to the best modern sanitary practice of quarantine was being enforced to prevent the cholera poison obtaining a foothold upon our shores.

But it must not be forgotten that there is no legal obligation on the part of Dr. Smith or of any other health officer to take such action in the interest of any but his own port and territory.

In this connection it is proper to state that Dr. Montizambert, in charge of the Grosse Ile quarantine below Quebec, writes the Secretary under recent date, forwarding a printed copy of the revised quarantine rules and regulations now enforced on the St. Lawrence, from which it is seen that the recommendations—as to quarantine practice, equipment, appliances, etc.—made in the report already referred to have been substantially adopted and carried out. This action materially increases the efficiency of one of the important safeguards of the interior, and there is no impropriety in citing it as one of the direct results of the quarantine inspection authorized by the Board in 1885.

In view of the manifestations of this pestilence during the past few months upon the Italian peninsula and in the Mediterranean, and the as yet uncontradicted assertion that it has re-appeared in South America, everything pertaining to our coast defenses against introduction of the disease is of very vital interest.

[During the session of the Board, information was received of the development of four cases of cholera upon the *Britannia*, a sister steamer of the *Alesia*. She arrived in New York bay on the 13th, and was detained at the upper quarantine station for observation until the 18th, when the disease was positively indentified, and the vessel was at once taken down to the lower station to be treated as the *Alesia* was.

This second arrival suggests the danger threatening the country from vessels sailing from ports of the infected Mediterranean to American ports, where no facilities exist for quarantine of observation, issolation, etc.]

The outbreak of yellow fever at Tampa and elsewhere in Florida have fortunately occurred too late in the season to give rise to much solicitude. The chief danger lies in the want of efficient health organizations throughout the territory affected, and the consequent probability of neglect to properly deal with infected material and localities. Such neglect may give rise to other outbreaks, or even an epidemic next year.

Although the public health of Illinois and the country generally has remained in a fairly satisfactory state, conditions obtained during the past summer, which caused grave solicitude. The intensely hot weather of July and the prolonged drought were among the more important of these. The great prevalence of diarrhœal diseases and numerous outbreaks of typhoid fever —the former directly due to the high temperature, and the latter intensified by water supplies, affected by the drought—were ominous of the results which might be anticipated if the specific poison of Asiatic cholera should be introduced. Fortunately this calamity was escaped, and the approach of cold weather assures us a further season of probable immunity from a cholera epidemic.

The only other noteworthy feature of the past quarter in this relation has been an extension of diphtheria—in many cases very malignant and fatal.

